# Development of a core outcome set for the evaluation of interventions to prevent COVID-19 in care homes (COS-COVID-PCARE Study)

**DOI:** 10.1186/s12877-022-03395-8

**Published:** 2022-08-27

**Authors:** Victoria Shepherd, Ishrat Islam, Fiona Wood, Paula R. Williamson, Claire Goodman, Philip M. Bath, Carl Thompson, Martin Knapp, Adam L. Gordon, Kerenza Hood

**Affiliations:** 1 Centre for Trials Research, Cardiff University, 4th floor Neuadd Meirionnydd, Heath Park, CF14 4YS Cardiff, UK; 2 PRIME Centre Wales, Wales, UK; 3 Division of Population Medicine, Cardiff University, Cardiff, Wales UK; 4 Clinical Trials Research Centre, University of Liverpool, Liverpool, UK; 5 Centre for Research in Public Health and Community Care (CRIPACC, University of Hertfordshire, Hatfield, UK; 6 NIHR Applied Research Collaboration (ARC) East of England Cambridge, Cambridge, UK; 7 Stroke Trials Unit, Mental Health & Clinical Neuroscience, University of Nottingham, Nottingham, UK; 8 School of Healthcare, Faculty of Medicine and Health, University of Leeds, Leeds, UK; 9 Care Policy and Evaluation Centre, London School of Economics and Political Science, London, UK; 10 Unit of Inflammation, Injury and Recovery Sciences, School of Medicine, University of Nottingham, Nottingham, UK; 11 NIHR Applied Research Collaboration-East Midlands (ARC-EM), Nottingham, UK

**Keywords:** Care homes, COVID-19, Pandemic, Prevention, Core outcome set

## Abstract

**Background:**

People living in care homes have experienced devastating impact from COVID-19. As interventions to prevent the transmission of COVID-19 are developed and evaluated, there is an urgent need for researchers to agree on the outcomes used when evaluating their effectiveness. Having an agreed set of outcomes that are used in all relevant trials can ensure that study results can be compared.

**Objective:**

The aim of the study was to develop a core outcome set (COS) for trials assessing the effectiveness of pharmacological and non-pharmacological interventions for preventing COVID-19 infection and transmission in care homes.

**Methods:**

The study used established COS methodology. A list of candidate outcomes was identified by reviewing registered trials to evaluate interventions to prevent COVID-19 in care homes. Seventy key stakeholders participated in a Delphi survey, rating the candidate outcomes on a nine-point scale over two rounds, with the opportunity to propose additional outcomes. Stakeholders included care home representatives (*n* = 19), healthcare professionals (*n* = 20), people with personal experience of care homes (*n* = 7), researchers (*n* = 15) and others (*n* = 9). Outcomes were eligible for inclusion if they met an a priori threshold. A consensus meeting with stakeholders resulted in agreement of the final outcome set.

**Results:**

Following the Delphi and consensus meeting, twenty-four outcomes were recommended for inclusion. These are grouped across four domains of infection, severity of illness, mortality, and ‘other’ (intervention specific or life impact). Due to the considerable heterogeneity between care homes, residents, and interventions, the relevance and importance of outcomes may differ between trial contexts. Intervention-specific outcomes would be included only where relevant to a given trial, thus reducing the measurement burden.

**Conclusion:**

Using a rapid response approach, a COS for COVID-19 prevention interventions in care homes has been developed. Future work should focus on identifying instruments for measuring these outcomes, and the interpretation and application of the COS across different trial contexts. Beyond COVID-19, the outcomes identified in this COS may have relevance to other infectious diseases in care homes, and the rapid response approach may be useful as preparation for future pandemics.

**Supplementary Information:**

The online version contains supplementary material available at 10.1186/s12877-022-03395-8.

## Background

COVID-19 has had devastating impact on people living in care homes [1]. In the UK, care homes are defined as long-term care facilities that provide accommodation together with personal or nursing care [2] which can be categorised as care homes with nursing provision (often termed nursing homes) and those without nursing (often termed residential homes) [3]. Older people are at very high risk of adverse outcomes from COVID-19 illness due to comorbidities associated with ageing and decreased immunological competence [4], and people with a learning disability are also at significantly increased risk of death due to COVID-19 although the causes are much less clear [5]. Preventing COVID-19 infection in care home residents depends on a hierarchy of control measures including vaccination of residents and staff, personal protective equipment (PPE), environmental modification, staffing arrangements, regular testing, and procedures for isolation and quarantine [6]. However, some of these restrictive measures risk harming the physical and mental wellbeing of care home residents [7], their families [8] and staff [9].

Developing effective interventions to prevent COVID-19 transmission in this susceptible population is the target of urgent public health research. Pharmacological interventions to prevent COVID-19 infection and transmission in care homes are in the early stages of testing [10]. Trials evaluating such interventions must choose appropriate outcome measures, defined as measures or observations used to capture and assess the consequences of treatment or support for individuals, such as assessment of side-effects (risk) or effectiveness (benefits) [11]. Outcome measure selection can be complex in these trials due to the range of potential outcomes and uncertainty surrounding a novel disease. Key criteria for outcome selection include responsiveness to the intervention and importance and acceptability to relevant stakeholders [11]. A core outcome set (COS) is a minimum set of outcomes that seeks to reduce heterogeneity of outcome reporting across trials, support meta-analyses of different studies and facilitate the synthesis of future research [11].

A number of COS have been developed in response to COVID-19 [12–14], including for COVID-19 prevention (COS-COVID-P) [15]. COS-COVID-P was developed to be applicable to all interventions and all care settings, with a need to develop additional COS for more specific settings noted. As interventions and outcome assessment will be different in care homes, a context-specific set of core outcomes for the prevention of transmission of COVID-19 in care homes is required. Key distinctions between care homes and hospitals or community settings include:

- higher risk of COVID-19 transmission, morbidity and mortality among care home residents [16]
- complex transmission routes due to communal living, staff-related factors (e.g., use of agency staff who move between care homes), and transfer between care homes and other settings, e.g. hospital [17]
- COVID-19 presents atypically in care home residents [18]
- challenges around reporting symptoms in a population where around 70% have cognitive impairment [19]
- a range of prevention strategies, guidance and policies designed and targeted specifically for care homes [20]
- potential benefits of prevention interventions (e.g., isolation measures) may be outweighed by their potential harms [20]

Building on an over-arching COVID-19 prevention core outcome set (COS-COVID-P), our aim in this study was to develop a specific COS ‘module’ for pharmacological and non-pharmacological interventions preventing COVID-19 infection and transmission in care homes (COS-COVID-PCARE).

## Methods

**Fig. 1:**
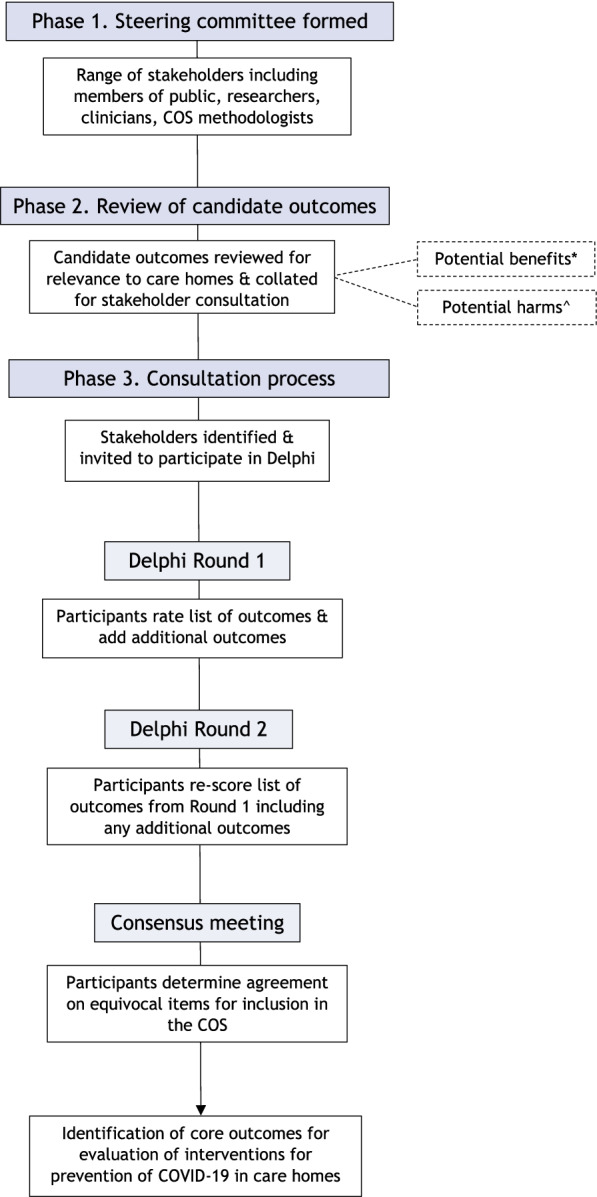
COS-COVID-PCARE development process. *^Whilst the potential benefits of prevention interventions may be common across these populations and settings (e.g., low rates of infection), there may be considerable differences between the potential harms of different types of interventions (e.g., isolation measures), and some harms may be specific or of greater importance to sub-populations of care homes and/or residents (social isolation and people living with dementia and dependent on others, for example)

The rapid pace of development of prevention and treatment strategies for COVID-19 necessitated a rapid response approach to COS development (see Fig. 1) building on the development of the over-arching COS-COVID-P. The COS was developed in accordance with the approach established by the COMET (Core Outcome Measures in Effectiveness Trials) initiative [11]. It is registered on the COMET database [21] and reported according to COS-STAR guidelines [22].

### Phase 1. Establishing a COS-COVID-PCARE steering committee

A steering committee was formed including members of the COS-COVID-P steering group, representatives from COMET [23], care home and care home research communities, and members of the public to ensure that the voices, views and experiences of people living in care homes (older people and people with a learning disability) and those who care for them were included. Committee members were identified through previous care home studies with the core project team and relevant research networks (see Acknowledgements section for details of members) and invited to join the virtual meetings held regularly during the study.

### Phase 2. Generating and reviewing candidate outcomes

The aim of phase 2 was to generate a list of candidate outcomes to be included in the Delphi survey. The COS-COVID-P steering group had previously conducted a review of registered trials for the prevention of COVID-19 in November 2020 [15] using the Cochrane COVID-19 study register [24]. Data from trials conducted with care home populations were extracted. The range and type of COVID-19 interventions were rapidly expanding, therefore the searches were updated by the COS-COVID-PCARE team in February 2021 and additional searches were conducted (e.g., NIHR Urgent Public Health studies [25]). Studies involving care homes were extracted to generate a list of candidate outcomes and domains. Other relevant documents, including the WHO minimal common outcome measure set for COVID-19 clinical research [13], were reviewed.

### Phase 3. Delphi survey with stakeholder groups

#### Participant identification and recruitment

The Delphi survey was open to people with an interest in preventing COVID-19 in care homes. Key stakeholders were invited to participate in the online Delphi survey, including members of groups who represent people living in care homes (including older people and people with a learning disability), family members of people living in care homes, researchers who design and conduct research in care homes, care home managers and staff, healthcare professionals, and others with an interest in care homes such as third sector organisations. Participants were recruited through existing research networks (e.g., ENRICH Cymru network) and other routes including charities and professional groups and via social media. Ethical approval was obtained from Cardiff University School of Medicine Research Ethics Committee. A user guide designed using accessibility principles was available to support participation, although it is not known how widely this was used.

#### Data collection

The survey was managed using the COMET DelphiManager online tool [26]. After being provided with information and consenting to take part, participants registered and were provided with a unique ID number. Once registered, reminder emails were sent to non-responders at each round.

##### Round 1

In round 1, participants provided demographic data including geographical region, stakeholder group, and whether their main interest was in relation to older people, younger people with disabilities, or both. Providing information about their interest in care home residents and which stakeholder group they belonged to was considered confirmation that they were eligible to take part. No participants who wished to take part were excluded. Participants scored a list of outcomes using a 9-point scale, where a score of 1–3 was interpreted as having ‘limited importance’, 4–6 as ‘important but not critical’ and 7–9 as ‘critical’ to include. Each outcome had an accompanying definition to aid understanding.

Initial outcomes were extracted from trials conducted during the early phases of the pandemic. Participants were encouraged to propose additional relevant outcomes not included. These outcomes were reviewed by the research team to ensure they were distinct from those already listed and relevant to care homes. Outcomes deemed out of scope or scored as ‘not important’ by proposers were removed. The steering committee reviewed the proposed new outcomes to be carried forward to Round 2.

##### Round 2

In the second round, participants were presented with the list of outcomes, alongside the distribution of scores for each outcome and the participant’s previous score. Participants were asked to rescore all outcomes. If they had changed a score they were asked to comment on why.

#### Data analysis

Once scored, outcomes were categorised as reaching ‘consensus in’, ‘consensus out’ or ‘no consensus’. We originally defined ‘consensus in’ as being ≥ 70% scoring 7 to 9 AND < 15% scoring 1 to 3 in each stakeholder group in line with COS guidance [11]. However, membership of the stakeholder groups was uneven, and some were relatively small, therefore consensus in every stakeholder group was not achievable. To ensure that stakeholders contributing from under-represented groups, such as those with personal experience, were not over-shadowed in the final scoring, we revised the threshold for consensus. This protocol amendment was made without reference to the results. For the ‘consensus in’ sub-analysis, we looked at whether each item had reached a majority for all groups with consensus being defined as ≥ 70% scoring 7 to 9 as a total group AND ≥ 50% scoring 7 to 9 in each stakeholder group. ‘Consensus out’ was defined as < 50% scoring 7 to 9 as a total group AND < 70% scoring 7 to 9 in each stakeholder group. ‘No consensus’ remained as anything else with no new compelling reasons provided.

### Stakeholder consensus meeting

The final phase of the consultation was an online meeting with participants from the Delphi survey who had previously indicated a willingness to take part in the consensus meeting. The purpose was to reach consensus on equivocal items, i.e., exhibited no consensus following the Delphi, or where further consultation/discussion was required.

All registered attendees were sent a briefing summary which included items for discussion and those items already included in the COS following the Delphi survey, and an auto-generated report which contained their own scores from the Delphi survey. This gave attendees the opportunity to reflect on items for discussion ahead of time, informed by their own previous scoring where relevant. Participants discussed and voted on each item. Discussions were audio-recorded (with verbal permission given by participants) and reasons for or against inclusion of each outcome were noted. As power is not distributed equally in consensus meetings [11] we emphasised the importance of a diverse range of opinions and that all opinions were valued [27]. Participants voted to either include or exclude the item, with a threshold of 70% for the combined group needed for inclusion in the final COS.

## Results

### Phase 2. Review of candidate items

The search for registered COVID-19 prevention studies identified 13 trials (see Additional file 1 for the list of included trials). Outcomes used in the identified trials were extracted, generating a list of 25 unique outcomes divided across four domains: infection, severity of disease, mortality, and other (intervention-related) outcomes (see Table 2 later).

### Phase 3. Delphi survey with stakeholder groups

The survey was conducted between March and June 2021, with a total of 70 participants registered. Participant demographics are detailed in Table 1.

**Table 1:** Participant characteristics

	**Participants registered (*n* = 70)**	**Participated in round 1 (*n* = 64)**^a^	**Participated in round 2 (*n* = 41)**^b^
**Stakeholder Group**			
Care home provider, manager, or staff	19	15	11
Clinician or healthcare professional	20	19	9
Personal experience e.g., family member	7	7	6
Researcher involved in care home studies	15	15	12
Other (e.g., working in social care policy)	9	8	3^c^
**Country**			
England	37	32	17
Scotland	5	5	4
Wales	27	26	19
Other European country	1	1	1
**Main group of interest**			
Older people	53	50	33
Younger people with disabilities	1	0	1
More than one group	14	12	6
Other	2	2	1

^a^No. of participants includes those who registered and provided incomplete data in round 1 (*n* = 6). Data included in the analysis

^b^No. of participants includes those who registered and provided incomplete data in round 2 (*n* = 2). Data included in the analysis

^c^One participant from the ‘other’ group identified as both a healthcare professional and having personal experience

#### Round 1

In addition to scoring the 25 outcomes, there were 41 new additional outcomes proposed by participants during round 1. These were analysed by the research team and those deemed to be out of scope of the COS, or which had been scored as ‘of limited importance’ by the proposer themselves, were removed. This resulted in 10 additional outcomes being included in round 2 (see Table 2), all categorised as ‘other’.

**Table 2:** Delphi survey items by round 2 scores from the combined stakeholder group and status prior to the consensus meeting

Outcome item	Domain	Score 1–3 n (%)	Score 4–6 n (%)	Score 7–9 n (%)	Decision
COVID-19 infection	Infection	0	2 (5)	38 (93)	Consensus in
COVID-19 infection with symptoms	Infection	0	2 (5)	38 (93)	Consensus in
COVID-19 infection with no symptoms	Infection	2 (5)	1 (2)	37 (91)	Consensus in
Immunity against COVID-19	Other	0	3 (7)	36 (88)	Consensus in
Uptake of vaccination	Other	0	3 (7)	36 (88)	Consensus in
COVID-19 related death	Mortality	2 (5)	4 (9)	35 (86)	Consensus in
Recovery from COVID-19 infection	Severity of illness	2 (5)	5 (12)	34 (83)	Consensus in
Feasibility of the intervention^a^	Other	0	5 (12)	32 (78)	Consensus in
Hospitalisation	Severity of illness	1 (2)	6 (15)	33 (81)	Consensus in
Staff knowledge and awareness about infection control^a^	Other	1 (2)	6 (15)	32 (78)	Consensus in
COVID-19 negative	Infection	0	7 (17)	34 (83)	Consensus in
Admission to Intensive Care Unit (ICU)	Severity of illness	2 (5)	7 (17)	32 (78)	Consensus in
Compliance with infection prevention guidance	Other	0	7 (17)	32 (78)	Consensus in
Mental health effects from the intervention	Other	0	8 (19)	32 (78)	Consensus in
Side effects or safety concerns	Other	0	7 (17)	31 (76)	Consensus in
Acceptability of the intervention^a^	Other	0	8 (19)	29 (71)	Consensus in not reached in all stakeholder groups
Quality of life and well-being^a^	Other	0	8 (19)	31 (76)	Consensus in
Condition getting better^b^	Severity of illness	2 (5)	9 (22)	30 (74)	Consensus in
Condition getting worse^b^	Severity of illness	2 (5)	9 (22)	30 (74)	Consensus in not reached in all stakeholder groups
Needing treatment with oxygen	Severity of illness	1 (2)	9 (22)	30 (74)	Consensus in
Staff knowledge and awareness about vaccines^a^	Other	2 (5)	9 (22)	29 (71)	Consensus in
Organs not functioning as well as expected	Severity of illness	3 (7)	9 (22)	29 (71)	Consensus in
Reduced blood oxygen levels	Severity of illness	2 (5)	9 (22)	28 (68)	Consensus in
Ability for residents to receive visits in the care home and/or for residents to make visits out of the home^a^	Other	1 (2)	12 (30)	27 (66)	No consensus
Impact on wider community transmission of COVID-19^a^	Other	5 (12)	9 (22)	25 (61)	No consensus
Non-COVID-19 related death	Mortality	4 (10)	13 (32)	24 (59)	No consensus
Decline in cognitive function (not due to COVID-19 illness)^a^	Other	1 (2)	13 (22)	22 (54)	No consensus
Change in ability to engage in usual activities (not due to COVID-19 illness)^a^	Other	1 (2)	16 (39)	22 (54)	No consensus
Inflammatory response	Other	2 (5)	17 (42)	16 (39)	Consensus out not reached in all stakeholder groups
Mental health getting worse	Severity of illness	1 (2)	20 (49)	17 (42)	Consensus out not reached in all stakeholder groups
Access to allied health professionals^a^	Other	4 (10)	18 (44)	15 (37)	Consensus out
Decline in ability to engage in usual activities due to COVID-19 illness	Severity of illness	1 (2)	20 (49)	15 (37)	Consensus out
Reduced appetite or fluid intake	Other	1 (2)	23 (56)	14 (36)	Consensus out
Other secondary infections	Infection	1 (2)	26 (63)	11 (27)	Consensus out
Needing to consult a General Practitioner (GP)	Severity of illness	4 (10)	22 (54)	11 (27)	Consensus out

% of participants who registered and provided full or partial data in round 2 (*n* = 41)

^a^denotes additional item proposed by participants during Delphi survey

^b^COVID-19 condition

#### Round 2

When scores in round 2 were analysed as a combined group, there were 23 items which met the ‘consensus in’ criteria for inclusion in the core outcome set although, when analysed by stakeholder group, two items did not reach the criteria. Seven items met the ‘consensus out’ threshold when analysed as a combined group, however two items did not reach the criteria in all stakeholder groups. Five items did not reach consensus (‘no consensus’). This resulted in 21 items being included in the COS and nine equivocal items (five’no consensus’ items plus four not reaching the criteria across all stakeholder groups) which required further discussion. Round 2 scores and status are shown in Table 2.

### Consensus meeting

An online consensus meeting was held via a video-conferencing platform (Zoom) in July 2021. Twenty-three attendees registered and twelve attended the consensus meeting which was hosted by three members of the research team. Attendees indicated via a poll at the start of the meeting which of the stakeholder groups best reflected their main interest in the area. Attendees were researchers (*n* = 6), clinicians (*n* = 2), had personal experience (*n* = 2) or ‘other’ (*n* = 2). None of the four care home staff who registered were able to attend on the day due to unforeseen events exacerbated by the pandemic. Attendees at the consensus meeting voted on the nine equivocal items (see Table 2), with three reaching the threshold for inclusion in the final COS (see Table 3).

**Table 3:** Items included in the core outcome set following the Delphi survey and consensus meeting

Detecting infection		
**Outcome**	**Definition**	**Comments or examples**
COVID-19 negative	Negative antigen test for COVID-19	• Antigen testing at individual level or whole care home (e.g. number or proportion of residents and/or staff, or number of days COVID-19 negative) • Score 0 on WHO Clinical Progression Scale
COVID-19 infection	Positive antigen test for COVID-19	• Antigen testing at individual level or whole care home (e.g. number or proportion of residents and/or staff)
COVID-19 infection with no symptoms	Virological but no clinical evidence of COVID-19	• Antigen testing and clinical assessment • Score 1 on WHO Clinical Progression Scale
COVID-19 infection with symptoms	Virological and clinical evidence of COVID-19	• Antigen testing and clinical assessment • Score ≥ 2 on WHO Clinical Progression Scale
**Severity of illness**		
**Outcome**	**Definition**	**Comments or examples**
Recovery from COVID-19 infection	Signs of clinical recovery	• Clinical assessment
Hospitalisation	Moderate-severe COVID-19 disease requiring hospital admission	• Score ≥ 4 on WHO Clinical Progression Scale
Reduced blood oxygen levels	Low oxygen saturation levels according to assessment and treatment protocol	• SpO_2_ measurement^a^ • Score ≥ 5 on WHO Clinical Progression Scale
Needing treatment with oxygen	Requiring supplemental oxygen in accordance with assessment and treatment protocol	• Requiring oxygen therapy e.g. via mask or nasal prongs • Score ≥ 5 on WHO Clinical Progression Scale
Organs not functioning as well as expected	Evidence of single or multiple organ dysfunction	• Assessment E.g Murray score, multiple organ dysfunction score • Score ≥ 5 on WHO Clinical Progression Scale
Admission to Intensive Care Unit (ICU)	Severe COVID-19 disease requiring ICU admission	• Score ≥ 6 on WHO Clinical Progression Scale
Condition getting better	Signs of improvement in clinical course	• Clinical assessment
**Mortality**		
**Outcome**	**Definition**	**Comments or examples**
COVID-19 related death	Death attributable to COVID-19	• Clinical assessment of cause of death • Score 10 on WHO Clinical Progression Scale
Non-COVID-19 related death^c^	Death attributable to causes other than COVID-19	• Clinical assessment of cause of death • Score 10 on WHO Clinical Progression Scale
**Other (intervention specific)**		
**Outcome**	**Definition**	**Comments or examples**
Has immunity against COVID-19	Evidence of antiviral activity in accordance with assessment protocol	• Measurement of antiviral activity using appropriate assay e.g. number or proportion of residents and/or staff
Uptake of vaccination	Evidence of uptake of vaccination in accordance with recommended schedule	• E.g number or proportion of residents and/or staff who have received vaccination in accordance with recommended schedule
Feasibility of the intervention^b^	Evidence of the feasibility of the intervention	• Assessment of the feasibility of implementing the intervention
Compliance with infection prevention guidance	Evidence of compliance with IPC^$^ measures	• Assessment of compliance with IPC^$^ measures in place
Mental health effects from the intervention	Evidence of changes in mental health (including delirium) attributed to the intervention	• Clinical assessment of mental health status • E.g delirium assessment tool
Side effects or safety concerns	Adverse events reported	• Reported in accordance with reporting procedures • E.g number or rates of adverse events reported in residents and/or staff
Staff knowledge and awareness about vaccines^b^	Evidence of staff knowledge and awareness about importance and use of vaccines	• Assessed level of staff knowledge and awareness
Staff knowledge and awareness about infection control^b^	Evidence of staff knowledge and awareness about importance and use of IPC^d^ measures	• Assessed level of staff knowledge and awareness
**Other (life impact)**		
**Outcome**	**Definition**	**Comments or examples**
Quality of life and well-being^b^	Quality of life and/or well-being experienced by residents	• Assessment of health-related quality of life, or social care-related quality of life, or overall well-being • E.g measure of quality of life or well-being
Ability for residents to receive visits in the care home and/or for residents to make visits out of the home^bc^	Any restriction on the ability for residents to make and receive visits	• Assessment of IPC^d^ measures in place
Decline in cognitive function (not due to COVID-19 illness)^bc^	Decline in cognition attributable to causes other than COVID-19	• Clinical assessment of cognitive function • E.g cognitive assessment tool

^a^SpO_2_ = oxygen saturation

^b^denotes additional item proposed by participants during Delphi survey

^c^denotes item included following discussion at the consensus meeting

^d^*IPC* Infection prevention and control

Attendees from care homes unable to attend on the day were contacted and invited to provide feedback on face validity. No responses were received. Items included in the final COS following the Delphi and consensus meeting (*n* = 24) are listed in Table 3.

## Discussion

This project established the items to be included in a core outcome set (COS) for evaluating interventions to prevent transmission of COVID-19 in care homes. The majority of items (*n* = 13) concern clinical improvement and/or survival, with considerable overlap with items on the WHO ordinal scale for clinical improvement [13]. The remainder (*n* = 11) are considered to be intervention-specific or have a broader impact on life, including quality of life and well-being. Of note, all the outcomes included in the ‘Other ‘life impact'’ category did not come from interventions described in the literature but were proposed by participants during the survey, thus highlighting the restricted focus on morbidity and mortality by trials conducted during this period which predominantly evaluated treatment and vaccines for COVID-19. Care homes are diverse, with considerable heterogeneity between layout and care arrangements, [28] residents [29] and interventions. Consequently, even though this is a setting-specific COS, the relevance and importance of outcomes may differ between homes. Thus highlighting the importance of context assessment at the pre-intervention stage [30].

The choice and timing of outcome measures for care home-focused research will need to take account of the relevant benefits and harms of (co-)interventions to address different situations (such as a COVID-19 outbreak in the care home), variable vaccination rates and wider community prevalence [31]. For example, high vaccination rates in residents, staff and visitors may enable other infection control measures such as visiting restrictions and isolation and quarantine of residents and staff to be lifted.

A Cochrane review of non‐pharmacological interventions to prevent or reduce transmission of COVID-19 in care homes published after the COS identified a range of primary outcomes used in 22 studies, including infections, hospitalisations and deaths due to COVID‐19, outbreaks in long‐term care facilities, and adverse health effects [32]. Intervention-specific domains included in the Cochrane review, such as surveillance and contact-regulation measures, were not included in our outcomes. The authors found limited reliable evidence on adverse and other unintended consequences of the interventions. They argued that the intrusiveness and burden of some of these interventions on residents living in care homes with higher vaccination rates needed to be measured alongside the evidence of their effectiveness [32]. This COS captures a range of setting-specific measures, some of which had not been included in any of the COVID-19 prevention trials conducted in care homes at this point, but it is still likely that there will need to be researcher discretion in how they are applied and ongoing review as interventions and circumstances change.

### Strengths and limitations

The list of candidate outcomes identified in Phase 2 from searches of relevant trials were predominantly from trials evaluating treatment and vaccines for COVID-19, reflecting the trials that were registered during the early phase of the pandemic when the study was initiated. To ensure the COS reflected the most up to date situation regarding COVID-19 trials, participants in the Delhi survey in Phase 3 were asked to propose additional outcomes based on their broader and more recent experience.

In accordance with COS development guidance, consideration was given to the representativeness of the stakeholders included in the study, and the ability of people across the different groups to engage with the consensus process [11]. A range of expertise and perspectives was sought to ensure that the project takes full account of the health and social needs of residents, alongside their emotional wellbeing, and respects residents’ rights and wishes. However, there were challenges around involving care home residents, many of who may lack capacity to consent [19]. This was compounded by care home staff in the consensus group being unable to attend the meeting despite registering to attend. We cannot rule out the possibility that some perceptions of outcome relevance and importance – important components of outcome quality—were not captured by the Delphi process. Participants predominantly responded in relation to older people living in care homes. The applicability of the COS to younger adults with disabilities as a discrete population living in care homes is therefore unclear. The survey was only available online and in English due to the time-limited nature of the study and, common amongst Delphi surveys, there is a potential for attrition bias. Additionally, participants were almost exclusively from the UK. As long-term care provision and public policy relating to COVID-19 will differ between countries, the applicability of the COS to care homes outside the UK will need to be considered prior to use.

Whilst not all modified Delphi approaches contain a consensus meeting component, it is now a well-established component [33]. The format and process of the consensus meeting was designed to take account of the potential issues around power and communication and other barriers that may affect participation by some stakeholders [11]. Strategies to minimize the influence of power differentials between different stakeholders during the meeting included ensuring good preparation, anonymous voting, and facilitation during the discussions [34]. However, voting may have been influenced by the stronger voicing of opinions from others during the meeting. The inclusion criteria for the consensus meeting, where the option was to include or exclude with > 70% required for inclusion, was different to the Delphi survey and designed to be definitive.

### Implications for the use of the COS in practice

A COS represents the minimum that should be measured and reported in trials, although outcomes used in a particular trial may not be restricted to only those in the COS [11]. The outcomes included in this COVID-PCARE COS will require careful thought around the interpretation of domains and items and their definition in order to avoid differential collection of outcomes. For example, presentation and understanding of symptoms of COVID-19 infection (and hence case definitions) have differed between jurisdictions and evolved over the course of the pandemic. Delirium was only included in diagnostic criteria for COVID-19 relatively late in the pandemic, despite being a common presenting feature in care home residents. This means that those using the COS should specify the symptoms being included and take account of our evolving understanding of the condition [35].

It is also important to recognise that some outcomes such as ‘needing treatment with oxygen’ could be interpreted either as ‘receiving treatment with oxygen’ or ‘having blood oxygen levels low enough to require treatment with oxygen’. Care home residents may deteriorate to the point that oxygen therapy is needed but they may not wish to attend hospital, it might be deemed inappropriate, or they may not be able to access such therapy in their care setting [20]. Others such as ‘COVID-19 infection’ and ‘COVID-19 negative’ relate to differences between detection-based outcomes and clinical or disease-burden outcomes. Several of the outcomes are related, thereby reducing the measurement burden (e.g., COVID-19 and non-COVID-19 related deaths could be considered as one cause-specific mortality outcome). Similarly, intervention-specific outcomes, such as staff knowledge and awareness about vaccines, will only be relevant to those trials which focus on behavioural interventions.

Ensuring COS uptake can be challenging; however, a number of factors can support uptake, including involving future implementers as stakeholders in the development of the COS and developing an implementation plan [7]. The findings from this project will be disseminated through care home research networks, such as NIHR Enabling Research in Care Homes (ENRICH), in order to inform future trials.

### Implications for future research

Once a COS has been agreed (the what to measure), the next stage in COS development is to determine how the outcomes included in the set should be defined and measured [11] using relevant guidance [36]. Following the approach used in the development of this COS, the measures may be applicable across interventions and populations, or relevant only to specific interventions and/or sub-populations of people living in care homes. Any novel disease may require the development of condition- or domain-specific outcome measurement instruments, but there are additional measurement challenges associated with this context. This includes some outcomes having temporal and/or co-diagnostic components, or which require differentiation between the effects of (co-)interventions and COVID-19 itself, for example a decline in cognitive function that is a consequence of the intervention and not due to COVID-19 illness.

Interventions to prevent COVID-19 may also have an impact on infection rates in care homes of other diseases such as influenza. Therefore, as COVID-19 transitions towards endemicity, the COVID-PCARE COS may have relevance beyond outbreaks of COVID-19 and beyond COVID-19 itself. The importance of better preparation for future pandemic research for older people has been highlighted [37], therefore the rapid response approach used and outcomes identified in this COS will also be relevant to future pandemics.

## Conclusion

This study has developed a COS for use in trials assessing the effectiveness of interventions to prevent COVID-19 in care homes across domains of infection, severity of illness, and mortality, as well as intervention-specific outcomes and those that have a broader impact on life. This recognises that interventions to prevent COVID-19 are not without harm and may not be used in isolation. (Co-) interventions will give rise to a range of benefits and burdens for those living in care homes and those who care for them, and so trials must take into account the outcomes that matter most to these groups. Future work must focus on interpretation of the outcomes in different trial contexts, and determine the most appropriate methods for measuring the outcomes included in this COS.

## Supplementary Information

**Additional file 1:** **Appendix 1.** List of registered COVID-19 prevention studies and relevant outcomes used to inform the Delphi survey.

## Data Availability

The dataset generated and used in this study is available through submission of a data request to the Centre for Trials Research at https://www.cardiff.ac.uk/centre-for-trials-research/about-us/data-requests.
