# Supplementary material for: Development of a core outcome set for the evaluation of interventions to prevent COVID-19 in care homes (COS-COVID-PCARE Study)
Source: BMC Geriatr. 2022 Aug 27;22:710. doi: 10.1186/s12877-022-03395-8 (PMC9417927; doi:10.1186/s12877-022-03395-8)
Supplement: Supplementary file 1 — Additional file 1: Appendix 1. List of registered COVID-19 prevention studies and relevant outcomes used to inform the Delphi survey. [file 12877_2022_3395_MOESM1_ESM.docx]

**Appendix 1. List of registered COVID-19 prevention studies and relevant outcomes used to inform the Delphi survey**

| **Setting category** | **Trial title** | **Trial population(s)** | **Country** | **Trial registration identifier*** | **Link to trial website and/or protocol** | **Intervention description** | **Primary outcome (1)** | **Primary outcome (2)** | **Secondary outcomes** |
| --- | --- | --- | --- | --- | --- | --- | --- | --- | --- |
| Healthcare workers (including nursing home workers) | Will Hydroxychloroquine Impede or Prevent COVID-19 (WHIP COVID-19) | Healthcare Workers and First Responders, age ≥18 years or older | US only | NCT04341441 | <https://www.henryford.com/whip-covid-19>  <https://clinicaltrials.gov/ProvidedDocs/41/NCT04341441/Prot_001.pdf> | Drug: Hydroxychloroquine - Daily Dosing Drug: Hydroxychloroquine - Weekly Dosing Other: Placebo oral tablet | Clinical COVID-19 Disease with laboratory confirmation of infection | __ | SARS-CoV-2 infections, clinical COVID-19 disease without laboratory confirmation, serology (IgM and IgG) positivity, SARS-CoV-2 viremia, adverse events related to study medication or procedures, HCQ blood levels, immunologic and serologic markers associated with SARS-CoV-2 infection and COVID-19 and COVID-19 disease related ED visits and hospitalizations. |
| Elderly Residents of Long-Term Care Facilities | Trial to Evaluate the Efficacy and Safety of Nitazoxanide (NTZ) for Post-Exposure Prophylaxis of COVID-19 and Other Viral Respiratory Illnesses in Elderly Residents of Long-Term Care Facilities (LTCF) | Resident in LTCFs at least 55 years of age | US only | NCT04343248 | __ | Drug: Nitazoxanide Drug: Placebo Dietary Supplement: Vitamin Super B-Complex | Symptomatic laboratory-confirmed COVID-19 [Time Frame: up to 6 weeks] | Symptomatic laboratory-confirmed VRI [Time Frame: up to 6 weeks] | __ |
| Nursing homes | A Study of LY3819253 (LY-CoV555) and LY3832479 (LY-CoV016) in Preventing SARS-CoV-2 Infection and COVID-19 in Nursing Home Residents and Staff (BLAZE-2) | Resident or facility staff in a skilled nursing or assisted living facility with at least one confirmed case of SARS-CoV-2 detection less than or equal to (≤)7 days prior to randomization | US only | NCT04497987 | <https://blaze2study.com/> | Drug: LY3819253 (Bamlanivimab) Drug: Placebo Drug: LY3832479 (Etesevimab) | Percentage of Participants with COVID-19 within 21 Days of Detection [Time Frame: Baseline through Week 8] | __ | Percentage of Participants with SARS-CoV-2, Moderate or Worse Severity COVID-19, hospitalisation, Emergency Room Visit, or Death, PK |
| Nursing homes | COVID-19 Project ECHO for Nursing Homes: A Patient-centered, Randomized-controlled Trial to Implement Infection Control and Quality of Life Best Practice | Skilled Nursing Facility | US only | NCT04499391 | <https://hsc.unm.edu/echo/institute-programs/covid-19-response/> | Project ECHO (an evidence-based telehealth model 16 weekly telehealth sessions addressing COVID-19 guidelines and best practices) ECHO Plus (an additional 9 sessions with a focus on CDC infection control training) | Infection rate [Time Frame: 2 years] | __ | Hospitalization [Time Frame: 2 years]  Death [Time Frame: 2 years] |
| Nursing homes | BCG Against Covid-19 for Prevention and Amelioration of Severity Trial (BAC to the PAST) | Residents of a participating LTCF 70 years or older | US only | NCT04534803 | __ | Drug: BCG Vaccine Other: Placebo | To assess the efficacy of BCG vaccination compared to placebo in reducing severe Covid-19 disease among elderly residents of skilled nursing facilities. [Time Frame: 12 months] | __ | To assess the efficacy of BCG vaccination compared to placebo in reducing the following among elderly residents of skilled nursing facilities (by number of cases) [Time Frame: 12 months]  To assess the efficacy of BCG vaccination compared to placebo in reducing the following among elderly residents of skilled nursing facilities (by number of episodes) [Time Frame: 12 months]  To assess the efficacy of BCG vaccination compared to placebo in reducing the following among elderly residents of skilled nursing facilities (by number of days) [Time Frame: 12 months] |
| Nursing home (elderly and health professionals) | Prevention of COVID19 Infection in Nursing Homes by Chemoprophylaxis With Hydroxychloroquine (PREVICHARM) | Nursing home residents and Healthcare professionals who provide direct care | Spain only | NCT04400019 | __ | Drug: Hydroxychloroquine Only Product in Oral Dose Form | Incidence of secondary cases of SARS-CoV-2 infection in residents and professionals in the 4 weeks after the start of the trial | __ | The effects on reduction in mortality and hospital admissions, incidence of symptoms, and drug safety will also be evaluated. |
| Long term care homes (LTCH) | Control of COVID-19 Outbreaks in Long Term Care | Residents 65 Years and older and staff in LTCH with outbreak of COVID-19 declared on at least one nursing unit | Canada only | NCT04448119 | __ | Drug: Favipiravir Drug: Placebo  As chemoprophylaxis: pre-exposure prophylaxis, post-exposure prophylaxis, pre-emptive therapy, or treatment for established COVID-19 | Control of Outbreak | __ | Effects on mortality, further infections in residents and staff, hospitalisations in residents, medical discontinuation due to adverse events in residents and staff, and new infections in other units of the facility. |
| Hospital settings, in outpatient care settings or in geriatric long-term care facilities | Chemoprophylaxis of SARS-CoV-2 Infection (COVID-19) in Exposed Healthcare Workers (COVIDAXIS) | Adult healthcare workers (HCWs) (for instance physicians, nurses, assistant nurses, dentists, physiotherapists, midwives, etc.) involved at the time of enrolment in the care and the management of patients with confirmed or suspected SARS-CoV-2 infection in hospital settings, in outpatient care settings or in geriatric long-term care facilities | France only | NCT04328285 | __ | Drug: Hydroxychloroquine Drug: Placebo of Hydroxychloroquine Drug: Lopinavir and ritonavir Drug: Placebo of LPV/r Tablets | Occurrence of an symptomatic or asymptomatic SARS-CoV-2 infection among healthcare workers (HCWs) [Time Frame: Up to 2.5 months] | __ | Effects on adverse events in each arm, discontinuation of medication, adherence, symptomatic cases, asymptomatic cases, severe infection, and safety. |
| Nursing home | COVID-19 Prophylaxis with hydroxychloroquine, Vitamin D, and Zinc supplementation in Danish nursing home residents – a randomized controlled trial | Nursing home residents ≥ 65 years of age | Denmark only | 2020-001363-85 | <https://www.clinicaltrialsregister.eu/ctr-search/trial/2020-001363-85/DK> | Drug: Hydroxychloroquine Drug: vitamin D Drug: zinc | To investigate whether prophylactic treatment with daily hydroxychloroquine, vitamin D, and zinc supplementation reduces the risk of COVID-19, the severity of the disease, the hospitalization rate, and death in nursing home residents. | SARS-CoV-2 positive rtPCR from nasopharyngeal samples during 2 months prophylactic treatment with hydroxychloroquine, vitamin D, and zinc supplement or no prophylactic treatment | To assess the effect on SARS-CoV-2 infection, influenza infection, Hospitalization, Need for intensive care respiratory support if hospitalized, Length of disease course, GP Contact, Side effects, and Death (30 days, 90 days) |
| Elderly in long-term and specialized care | COVID-19 PEP- High-risk Individuals in Long-term and Specialized Care - Canada | Patient/resident in an Institute (to include a rehabilitation, long term care facility, mental health facility or veteran's care) that provides bed-based care in shared semi-private or ward rooms (i.e. two or more to a room) with a patient with confirmed COVID-19 for at least 6 hours in the absence of contact and droplet precautions. | Canada only | NCT04397328 | __ | Drug: Hydroxychloroquine Drug: Placebo | Symptomatic infection [Time Frame: 90 days after exposure] | __ | Hospital and/or ICU admission or death, time to recovery |
| Nursing home | Training the Innate Immune System Against SARS-CoV-2 (COVID-19) Using the Shingrix Vaccine in Nursing Home Residents (NH-Shingrix) | Nursing home residents ≥ 65 years of age | US only | NCT04523246 | __ | Drug: SHINGRIX (Zoster Vaccine REcombinant, Adjuvanted) | Evidenced of active and trained innate immunity [Time Frame: Day 91 and 120 (post vaccination)] | __ | Respiratory Disease Severity (6 month) [Time Frame: Days 120 through 180] |
| Nursing home | COVID-FIS: A Phase 2 Placebo-Controlled Pilot Study in COVID-19 of Fisetin to Alleviate Dysfunction and Excessive Inflammatory Response in Older Adults in Nursing Homes | Nursing home residents ≥ 65 years of age | US only | NCT04537299 | __ | Drug: Fisetin  Dose: ~20 mg/kg/day oral, NG or D tube course for 2 consecutive days twice (Days 0 & 1 and Days 8 & 9) | Change in COVID-19 Severity [Time Frame: baseline, Day 2, 7, 10, 14, 17, 30, 90 and 180]  Ordinal Scale for Clinical Improvement (minimum=0 and maximum=8; higher score = worse outcome) | __ | __ |
| Care homes | Best Practices to Prevent COVID-19 Illness in Staff and People With Serious Mental Illness and Developmental Disabilities in Congregate Living Settings | Residents and staff of the home ≥ 18 years of age | US only | NCT04726371 | __ | Tailored Best Practices (TBP) intervention package as part of routine training activities. TBP consists of COVID-19 mitigation measures specifically adapted for staff and residents with SMI and ID/DD in congregate living settings | New COVID-19 Group Home Incidence at Baseline [Time Frame: baseline and 3, 6, 12, 15 months] | Best practice fidelity [Baseline and 3, 6, 12, 15 months] | __ |

*Registration number e.g ClinicalTrials.gov, EudraCT
